# Noninvasive Estimation of Joint Moments with Inertial Sensor System for Analysis of STS Rehabilitation Training

**DOI:** 10.1155/2018/6570617

**Published:** 2018-02-11

**Authors:** Kun Liu, Jianchao Yan, Yong Liu, Ming Ye

**Affiliations:** School of Mechanical Science and Engineering, Jilin University, Jilin 130025, China

## Abstract

An original approach for noninvasive estimation of lower limb joint moments for analysis of STS rehabilitation training with only inertial measurement units was presented based on a piecewise three-segment STS biomechanical model and a double-sensor difference based algorithm. Joint kinematic and kinetic analysis using a customized wearable sensor system composed of accelerometers and gyroscopes were presented and evaluated compared with a referenced camera system by five healthy subjects and five patients in rehabilitation. Since there is no integration of angular acceleration or angular velocity, the result is not distorted without offset and drift. Besides, since there are no physical sensors implanted in the lower limb joints based on the algorithm, it is feasible to noninvasively analyze STS kinematics and kinetics with less numbers and types of inertial sensors than those mentioned in other methods. Compared with the results from the reference system, the developed wearable sensor system is available to do spatiotemporal analysis of STS task with fewer sensors and high degree of accuracy, to apply guidance and reference for rehabilitation training or desired feedback for the control of powered exoskeleton system.

## 1. Introduction

Sit-to-stand (STS) movement is one of the most commonly performed functional activities [1, 2], which requires both relatively large joint moments and precise balance control [3, 4]. STS movement is also a complex dynamic task that requires regulation of lower limb muscles to drive the human body while rising from a chair from a stable seated position to a relatively unstable upright stance [5–8]. However, for dependent people having lost part of their lower limb functionalities without adequate joint moment, the activity becomes tiring and cannot be accomplished without the help of external assistance [9]. Therefore, the ability to perform STS transfer in a reliable and safe manner with adequate joint moment [10] becomes a key element of movement rehabilitation in orthopedically or neurologically impaired individuals.

Ambulatory estimation and analysis of STS movement with wearable sensors is a promising clinical tool to diagnose human motion. Quantitative kinetic and kinematic information of STS is crucial for the clinical evaluation and therapeutic treatment comparisons in the orthopedic and rehabilitation fields. However, since the camera-based human motion analysis system is bulky, expensive, and complex, it restricts the user to a constrained environment where cameras are installed; therefore, it is not applicable for out-lab ambulatory measurement of lower limb posture in ordinary life [11]. Recently, small inertial sensor measurement modules (IMUs) comprised of accelerometers, gyroscopes, and (or) magnetometers were developed and appear to be promising for estimating human movement. Various methods using inertial sensors were available for assessing 3D human posture in motion [12–14]; however, few papers proposed methods to estimate kinetics of lower limb joints using wearable inertia sensors and also few detailed applications of inertia sensors for noninvasive analysis and diagnosis of STS rehabilitation training.

In order to make full use of the remaining muscle power of the patient, assistant systems such as exoskeleton/orthosis and partial body weight support (PBWS) rehabilitation robot were designed to make up for the lack of joint moment with external assistance [15–18]. One of the key technical problems of assistant system is how to noninvasively estimate the joint moment with wearable sensors especially for the control of wearable exoskeleton/orthosis. Furthermore, monitored rehabilitation exercises have been shown to be more effective than practice without feedback [19]. Thence, the wearable monitoring of any STS rehabilitation program is desirable to ensure the correct execution of the exercise by patients and also to quantify the progress toward the recovery of muscle strength, endurance, and increase in the range of motion. Bonnet et al. [20] investigated the possibility of estimating 3D lower limb joint kinematics using a single inertial measurement unit during lower limb rehabilitation. But they only monitored the hip and knee joint angles without any kinetic analysis of human motion. Stieglitz et al. [21] designed a setup to noninvasively measure the joint torque development at given ankle positions in an intact leg, but it is only applicable to test the isometric torque development in accordance to the anatomical features of the rat model, not a human biomechanical model. Inkster et al. [22] analyzed the joint moment for postural control during STS task by individuals with mild Parkinson's disease. But all of the kinematic and kinetic data were obtained from nonwearable imaging systems and force plate in the lab, which were only suitable for offline processing and analysis. Yoshioka et al. [23, 24] did biomechanical kinematic and kinetic analyses of STS movements and computed minimum peak joint moments and analyzed the relation between movement time and joint moment development during a STS task. Although their presented research has practical applications in STS rehabilitations and exercise prescription, the required muscle strength was quantitatively estimated based on optical motion capture system with 7 cameras, which occupied a lot of space and was not convenient for portable systems with real-time control. Wang et al. [25] presented a method to predict the joint moment using wearable EMG sensors with a neural network model of muscle activations, but it was validated at the elbow, not the lower limb joints. Karatsidis et al. [26] demonstrated that estimation of 3D ground reaction force and moments during gait using only kinematic information obtained from inertial sensors agreed with “gold-standard” force plate measurements, but there was no further analysis of the joint kinetics.

The purpose of this study is to noninvasively estimate the joint moments using wearable IMUs to provide reference for making exercise prescription of STS rehabilitation training. The kinematic and kinetic profiles underlying the STS movement were analyzed with two groups: (1) patients with lower limb muscle dysfunction but in rehabilitation and could perform STS task independently and (2) age-matched healthy controls that could perform in influent STS motion task independently. Compared with the healthy subjects, it was hypothesized that patients in STS rehabilitation would exhibit altered anticipatory postural control which would include an increased preparatory hip flexion and forward displacement of the COM prior to seat-off. By comparing with the kinematic and kinetic data derived from the IMUs and referenced camera system and force plate, the accuracy and availability of the developed wearable sensor system were verified. By comparing and analyzing the calculated joint moments of two groups of the subjects, the rehabilitation condition of the patients with lower limb muscle dysfunction during the STS training were more explicit. It has the extensive applicability and practical significance for making exercise prescription of STS rehabilitation training. Also, the presented method to noninvasively estimate the lower limb joint moments using IMUs was useful and crucial for the real-time control of wearable exoskeleton/orthosis.

## 2. Methods

**Figure 1:**
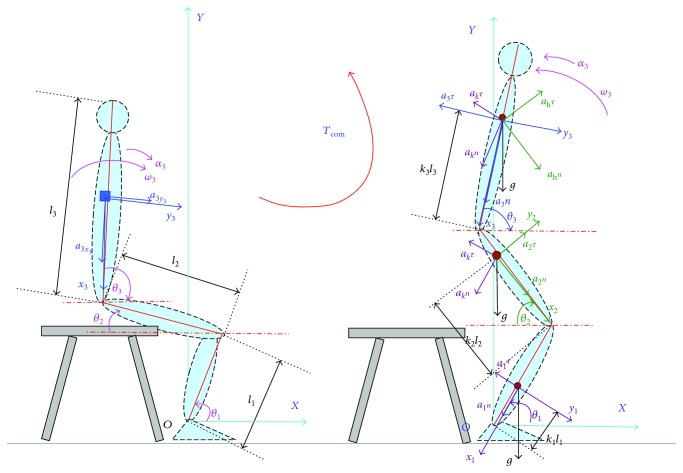
Kinematic analysis of STS movement in sagittal plane based on a piecewise 2D three-segment STS biomechanical model of the human body.

**Figure 2:**
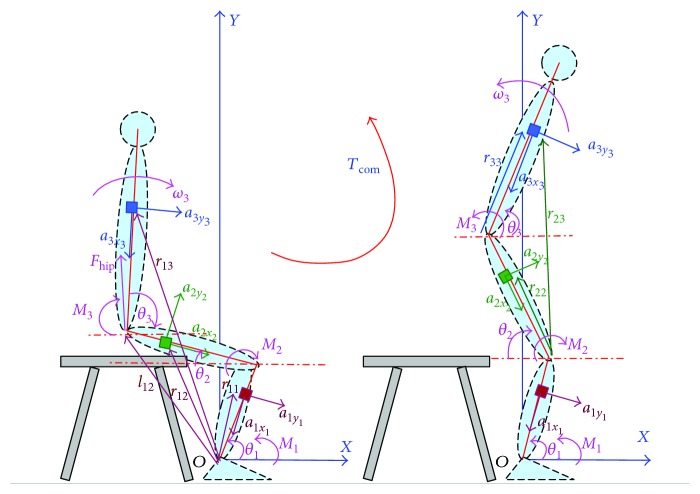
Kinetic analysis of STS movement in sagittal plane based on a piecewise 2D three-segment STS biomechanical model of the human body.

As we presented [27], the segment rotational angles (joint angles) in sagittal plane could be calculated using the sensor-measured accelerations based on a double-sensor difference based algorithm. Then the angular acceleration of each segment based on the calculated joint angles and measured accelerations was calculated. In order to do kinematic and kinetic analyses of STS movement using wearable IMUs, a piecewise two-dimensional (2D) three-segment STS biomechanical model of the human body is needed. A piecewise biomechanical model for STS kinematic analysis was presented in our previous work [28] just for a control strategy research, but there was no kinetic analysis of STS. Here the model is further developed for the STS joint kinetic analysis using IMUs as shown in Figure 1 and Figure 2. Then we did the kinetics and kinematics analysis of the STS movement and proposed the angular accelerations of the shank, thigh, and HAT (*α*_1_, *α*_2_, and *α*_3_) and the joint moments (*M_i_*, where *i* = 1, 2, 3) based on the further developed model of that which we presented in our previous work [28]

Before the buttocks leaved the chair (seat-off), the lower limb segment remained stationary, and the main movement was that the HAT (head, neck, arms, and torso) rotated forward around the hip joint with a certain spinal curvature. That the spinal curvature would lead to length variation of the HAT and location change of the COM of HAT will make the calculated result deviate from the true value. Therefore, in the experiment, the subjects would be told to minimize the spinal curvature as possible as they could and the spinal curvature was not taken into account in the STS biomechanical model. Therefore, based on the piecewise 2D three-segment STS biomechanical model as proposed in Figure 1, we calculated the angular acceleration (*α*_3_) of center of mass (COM) of the HAT before seat-off.

(1) $$\begin{array}{c} \alpha_{3}={\ddot{\theta }}_{3}=\frac{g\cos\theta_{3}-a_{3y_{3}}}{k_{3}l_{3}}. \end{array}$$

After seat-off as shown in Figure 1, the three segments of the whole body rotated about their corresponding joints until the whole body stretched to the upright static posture finally. We calculated the angular accelerations of the shank, thigh, and HAT (*α*_1_, *α*_2_, and *α*_3_) based on the calculated joint angles and angular velocities as shown in

(2) $$\begin{array}{c} \alpha_{1}={\ddot{\theta }}_{1}=k_{1}{l_{1}}^{-1}\left(g\cos\theta_{1}-a_{1y_{1}}\right),\\ \alpha_{2}={\ddot{\theta }}_{2}={\left(k_{2}l_{2}\right)}^{-1}\left(a_{2y_{2}}+l_{1}\omega_{1}^{2}\sin\left(\theta_{1}+\theta_{2}\right)-k_{1}^{-1}\left(g\cos\theta_{1}-a_{1y_{1}}\right)\cos\left(\theta_{1}+\theta_{2}\right)+g\cos\theta_{2}\right),\\ \alpha_{3}={\ddot{\theta }}_{3}={\left(k_{3}l_{3}\right)}^{-1}\left\{{k_{2}}^{-1}\left(a_{2y_{2}}+l_{1}\omega_{1}^{2}\sin\left(\theta_{1}+\theta_{2}\right)-{k_{1}}^{-1}\left(g\cos\theta_{1}-a_{1y_{1}}\right)\cos\left(\theta_{1}+\theta_{2}\right)+g\cos\theta_{2}\right)\cos\left(\theta_{2}+\theta_{3}\right)+a_{3y_{3}}-g\cos\theta_{3}+l_{1}\omega_{1}^{2}\sin\left(\theta_{3}-\theta_{1}\right)+{k_{1}}^{-1}\left(g\cos\theta_{1}-a_{1y_{1}}\right)\cos\left(\cos\theta_{3}-\theta_{1}\right)-l_{2}\omega_{2}^{2}\sin\left(\theta_{2}+\theta_{3}\right)\right\}, \end{array}$$

where *l_i_*, *i* = 1, 2, 3, is the length of the shank, thigh, and HAT, *θ_i_*, *i* = 1, 2, 3, is the ankle, knee, and hip joint angles which were calculated based on a double-sensor difference based algorithm [23], and *ω_i_*, *i* = 1, 2, 3, is the angular velocities of the shank, thigh, and HAT about the corresponding joint. *T*_com_ is the STS trajectory of COM.

Based on kinetic analysis of STS as shown in Figure 2, we calculated the joint moments (*M_i_*, *i* = 1, 2, 3) before seat-off as follows:

(3) $$\begin{array}{c} {\vec{M}}_{3}=J_{3}\cdot {\vec{\alpha }}_{3}+{\vec{r}}_{33}\times \left(m_{3}\cdot {\vec{a}}_{3}\right),\\ {\vec{M}}_{2}=J_{3}\cdot {\vec{\alpha }}_{3}+{\vec{r}}_{23}\times \left(m_{3}\cdot {\vec{a}}_{3}\right)+{\vec{r}}_{22}\times \left(m_{2}\cdot \vec{g}\right)-{\vec{l}}_{22^{\prime }}\times {\vec{F}}_{hip},\\ {\vec{M}}_{1}=J_{3}\cdot {\vec{\alpha }}_{3}+{\vec{r}}_{13}\times \left(m_{3}\cdot {\vec{a}}_{3}\right)+{\vec{r}}_{12}\times \left(m_{2}\cdot \vec{g}\right)+{\vec{r}}_{11}\times \left(m_{1}\cdot \vec{g}\right)+{\vec{l}}_{12^{\prime }}\times {\vec{F}}_{hip}. \end{array}$$

Then the joint moments before seat-off were reformulated in detail as follows:

(4) $$\begin{array}{c} M_{3}=J_{3}{\left(k_{3}l_{3}\right)}^{-1}\left(a_{3y_{3}}-{gc}_{3}\right)+m_{3}a_{3y_{3}}k_{3}l_{3},\\ M_{2}=F_{hip}l_{2}c_{2}-J_{3}{\left(k_{3}l_{3}\right)}^{-1}\left(a_{3y_{3}}-{gc}_{3}\right)-m_{2}{gk}_{2}l_{2}c_{2}-m_{3}a_{3x_{3}}{\left(2k_{3}l_{3}\right)}^{-1}\left(2l_{2}^{2}-2l_{2}k_{3}l_{3}c_{2+3}\right)-m_{3}a_{3y_{3}}{\left(2k_{3}l_{3}\right)}^{-1}\left(2k_{3}^{2}l_{3}^{2}-2l_{2}k_{3}l_{3}c_{2+3}\right),\\ M_{1}=J_{3}{\left(k_{3}l_{3}\right)}^{-1}\left(a_{3y_{3}}-{gc}_{3}\right)+m_{1}{gk}_{1}l_{1}c_{1}+F_{hip}\left(l_{2}c_{2}-l_{1}c_{1}\right)+m_{3}a_{3x_{3}}{\left(2k_{3}l_{3}\right)}^{-1}\left({\left(2l_{1}^{2}+2l_{2}^{2}+l_{3}^{2}-4l_{1}l_{2}c_{1+2}+2l_{1}k_{3}l_{3}c_{1-3}-2l_{2}k_{3}l_{3}c_{2+3}\right)}^{1/2}-k_{3}^{2}l_{3}^{2}\right)-m_{3}a_{3y_{3}}{\left(2k_{3}l_{3}\right)}^{-1}\left(k_{3}^{2}l_{3}^{2}+l_{3}^{2}+2l_{1}k_{3}l_{3}c_{1-3}-2l_{2}k_{3}l_{3}c_{2+3}\right). \end{array}$$

We also calculated the moments of the hip, knee, and ankle joint after seat-off as follows:

(5) $$\begin{array}{c} {\vec{M}}_{3}=J_{3}\cdot {\vec{\alpha }}_{3}+{\vec{r}}_{33}\times \left(m_{3}\cdot {\vec{a}}_{3}\right),\\ {\vec{M}}_{2}=-J_{3}\cdot {\vec{\alpha }}_{3}-J_{2}\cdot {\vec{\alpha }}_{2}-{\vec{r}}_{23}\times \left(m_{3}\cdot {\vec{a}}_{3}\right)-{\vec{r}}_{22}\times \left(m_{2}\cdot {\vec{a}}_{2}\right),\\ {\vec{M}}_{1}=J_{3}\cdot {\vec{\alpha }}_{3}+J_{2}\cdot {\vec{\alpha }}_{2}+J_{1}\cdot {\vec{\alpha }}_{1}+{\vec{r}}_{13}\times \left(m_{3}\cdot {\vec{a}}_{3}\right)+{\vec{r}}_{12}\times \left(m_{2}\cdot {\vec{a}}_{2}\right)+{\vec{r}}_{11}\times \left(m_{1}\cdot {\vec{a}}_{1}\right). \end{array}$$

Then the joint moments after seat-off were reformulated in detail as follows:

(6) $$\begin{array}{c} M_{3}=J_{3}{\left(k_{3}l_{3}\right)}^{-1}\left({k_{2}}^{-1}\left(a_{2y_{2}}+l_{1}\omega_{1}^{2}s_{1+2}-{k_{1}}^{-1}\left({gc}_{1}-a_{1y_{1}}\right)c_{1+2}+{gc}_{2}\right)c_{2+3}+a_{3y_{3}}-{gc}_{3}+l_{1}\omega_{1}^{2}s_{3-1}+{k_{1}}^{-1}\left({gc}_{1}-a_{1y_{1}}\right)c_{3-1}-l_{2}\omega_{2}^{2}s_{2+3}\right)+m_{3}a_{3y_{3}}k_{3}l_{3},\\ M_{2}=-J_{2}{\left(k_{2}l_{2}\right)}^{-1}\left(a_{2y_{2}}+l_{1}\omega_{1}^{2}s_{1+2}-{k_{1}}^{-1}\left({gc}_{1}-a_{1y_{1}}\right)c_{1+2}+{gc}_{2}\right)-J_{3}{\left(k_{3}l_{3}\right)}^{-1}\left({k_{2}}^{-1}\left(a_{2y_{2}}+l_{1}\omega_{1}^{2}s_{1+2}-{k_{1}}^{-1}\left({gc}_{1}-a_{1y_{1}}\right)c_{1+2}+{gc}_{2}\right)c_{2+3}+a_{3y_{3}}-{gc}_{3}+l_{1}\omega_{1}^{2}s_{3-1}+{k_{1}}^{-1}\left({gc}_{1}-a_{1y_{1}}\right)c_{3-1}-l_{2}\omega_{2}^{2}s_{2+3}\right)-m_{2}a_{2y_{2}}k_{2}l_{2}-m_{3}a_{3x_{3}}{\left(2k_{3}l_{3}\right)}^{-1}\left(2l_{2}^{2}-2l_{2}k_{3}l_{3}c_{2+3}\right)-m_{3}a_{3y_{3}}{\left(2k_{3}l_{3}\right)}^{-1}\left(2k_{3}^{2}l_{3}^{2}-2l_{2}k_{3}l_{3}c_{2+3}\right),\\ M_{1}=J_{1}{\left(k_{1}l_{1}\right)}^{-1}\left({gc}_{1}-a_{1y_{1}}\right)+J_{2}{\left(k_{2}l_{2}\right)}^{-1}\left\{a_{2y_{2}}+l_{1}\omega_{1}^{2}s_{1+2}-{k_{1}}^{-1}\left({gc}_{1}-a_{1y_{1}}\right)c_{1+2}+{gc}_{2}\right\}+J_{3}{\left(k_{3}l_{3}\right)}^{-1}\left\{{k_{2}}^{-1}\left\{a_{2y_{2}}+l_{1}\omega_{1}^{2}s_{1+2}-{k_{1}}^{-1}\left({gc}_{1}-a_{1y_{1}}\right)c_{1+2}+{gc}_{2}\right\}c_{2+3}+a_{3y_{3}}-{gc}_{3}+l_{1}\omega_{1}^{2}s_{3-1}+{k_{1}}^{-1}\left({gc}_{1}-a_{1y_{1}}\right)c_{3-1}-l_{2}\omega_{2}^{2}s_{2+3}\right\}+m_{1}a_{1y_{1}}k_{1}l_{1}+m_{2}a_{2x_{2}}{\left(2k_{2}l_{2}\right)}^{-1}\left(2l_{1}^{2}-2l_{1}k_{2}l_{2}c_{1+2}\right)+m_{2}a_{2y_{2}}{\left(2k_{2}l_{2}\right)}^{-1}\left(2k_{2}^{2}l_{2}^{2}-2l_{1}k_{2}l_{2}c_{1+2}\right)+m_{3}a_{3x_{3}}{\left(2k_{3}l_{3}\right)}^{-1}\left({\left(2l_{1}^{2}+2l_{2}^{2}+l_{3}^{2}-4l_{1}l_{2}c_{1+2}+2l_{1}k_{3}l_{3}c_{1-3}-2l_{2}k_{3}l_{3}c_{2+3}\right)}^{1/2}-k_{3}^{2}l_{3}^{2}\right)-m_{3}a_{3y_{3}}{\left(2k_{3}l_{3}\right)}^{-1}\left(k_{3}^{2}l_{3}^{2}+l_{3}^{2}+2l_{1}k_{3}l_{3}c_{1-3}-2l_{2}k_{3}l_{3}c_{2+3}\right). \end{array}$$

As shown in all the equations, the joint moments were calculated from the sensor-measured data without any integral or differential operations.

The mass and dimension of each segment of the subjects were estimated based on the average current Chinese male inertial parameters of body segments according to Chinese national standards [29] as shown in Table 1. The moment of inertia of each segment was estimated based on the height and total mass of the subject [30] and shown in Table 1. All segments were assumed to be rigid, and the STS movement was performed only in the sagittal plane.

## 3. Experiment

Two force plates were developed with pressure sensors (YZC-1B) and sampled at 100 Hz using a microcontroller (Arduino UNO). One force plate (force plate A) was placed on the anterior section of the chair to measure the vertical chair reaction force (VCRF) before subjects' thighs left the seat (seat-off). Another force plate (force plate B) was fixed on the ground under subjects' feet to measure the vertical ground reaction force (VGRF) throughout the STS process. To measure the accelerations and angular velocities of the segments for calculating the joint angles, angular accelerations, and joint moments, three customized IMUs (wearable sensor JY-901B, 1.1 × 1.1 × 0.5 inches with battery and Bluetooth communication) were attached on the lateral surface of shank, thigh, and HAT with elastic straps, coinciding with the COM of each segment in the sagittal plane as shown in Figure 2 as possible. A microcontroller (Arduino UNO) was used to capture accelerations and angular velocity data from the IMUs at 100 Hz, store data, and communicate with a PC in real time. During the initial calibration, the two force plates were positioned horizontally under the feet and on the chair with calibration errors of 0.53% and 0.61% in the vertical direction. The orientation of segments was estimated by combining the orientations of individual IMUs with the STS biomechanical model of the human body. To relate the sensor orientations to segment orientations, a sensor-to-segment calibration procedure is performed referring to [26]. Each of the three IMUs was repeatedly adjusted in the sagittal plane with the *x*-axis coinciding with the axial direction of its corresponding segment in the segment coordinate frames based on the recommendations of the International Society of Biomechanics [31]. Simultaneously, a commercial optical motion analysis system, NAC Hi-Dcam II Digital High Speed Camera Systems (NAC Image Technology, Japan), was used to track and measure the 3D trajectories of the retroreflective markers on the segments of the subjects, with sampling frequency of 100 Hz and calibration error 0.22%. Then the referenced angle, angular velocity, and acceleration of the segments were obtained from the referenced camera system by analyzing the motion parameters of the markers and then were used to calculate the angular accelerations and joint moments based on the developed method offline.

**Figure 3:**
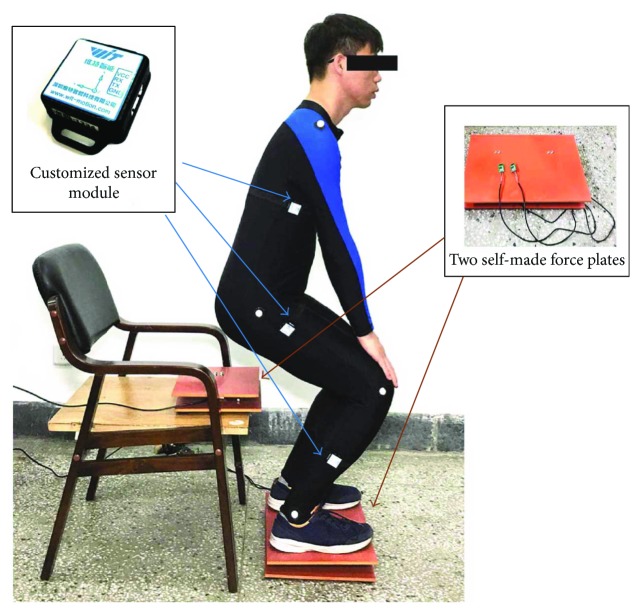
Experiment using the developed sensor system in the working space of the RCS.

Five healthy male subjects (age = 28.1 ± 6.3 years; mass = 67.3 ± 8.5 kg; height = 173.5 ± 6.7 cm) without known lower limb musculoskeletal or neurological dysfunction and five male patients in STS rehabilitation (age = 29.5 ± 7.5 years; mass = 65.4 ± 6.3 kg; height = 172.5 ± 7.6 cm) with mild lower limb dyskinesia but without affecting their ability of performing STS movement participated in this study by two groups and received informed consent. The experimental protocol was approved by the Human Ethical Review Committee of Jilin University. After familiarization, each of the ten participants reported no serious impediment of either IMUs or force plates and performed three STS trials as a task at self-selected appropriate speed (healthy subjects' STS within 3 s and patients' STS within 7 s) wearing the developed sensor system in the working space of the referenced optical motion capture system (RCS). Finally, 30 trials (3 trials × 1 task per subject × 10 subjects) were achieved for analysis. Although a task of three STS trials were performed by one subject, the STS time and the amount of captured data of each trial could not be absolutely the same. Therefore, the time of zero VCRF (time of seat-off) was so important that it was designated as the referenced standard point (RSP) within whole STS to synchronize the three trials of a task to the same percentage metric, then the ensemble averages of the captured angular velocities and accelerations of the three STS trails in a task performed by one subject were got for calculating the compositive joint angles and angular accelerations of each segment's COM in the STS movement. By comparing with the processed kinematic and kinetic data of the ten tasks achieved from the IMUs and RCS, the accuracy and availability of the developed wearable sensor system could be verified. Furthermore, the joint moments of ankle, knee, and hip joints of each subject of the ten were calculated based on the corresponding ensemble averages of all the original inertial parameters of a task derived from the sensor system. Five groups of hip, knee, and ankle joint moments of the five healthy subjects were, respectively, synchronized again to get a group of ensemble average joint moments as references for comparing and analyzing the STS rehabilitation conditions of the five patients.  Figure 3 shows a healthy male subject performing a STS trial with the developed sensor system in the working space of the RCS.

## 4. Result

**Figure 4:**
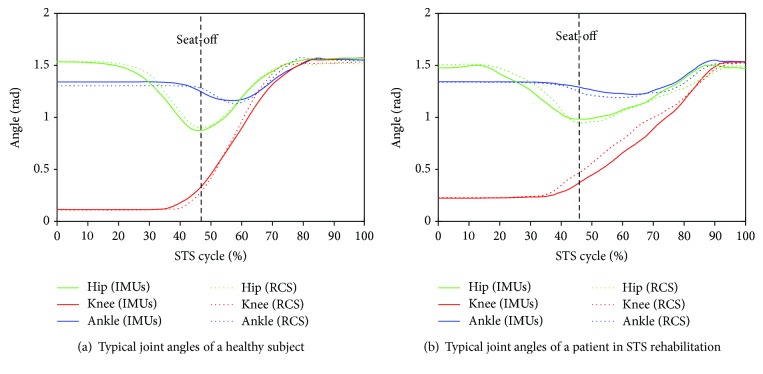
Typical hip, knee, and ankle joint angles during the STS tasks performed by a healthy subject and a patient.

**Figure 5:**
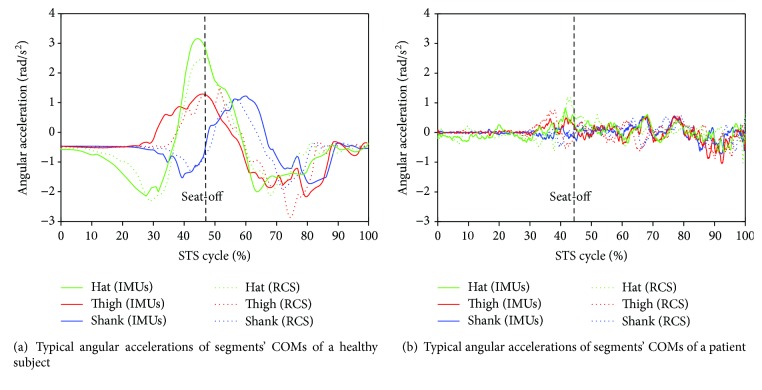
Typical angular accelerations of the COMs of HAT, thigh, and shank segments during the STS task performed by a healthy subject and a patient.

**Figure 6:**
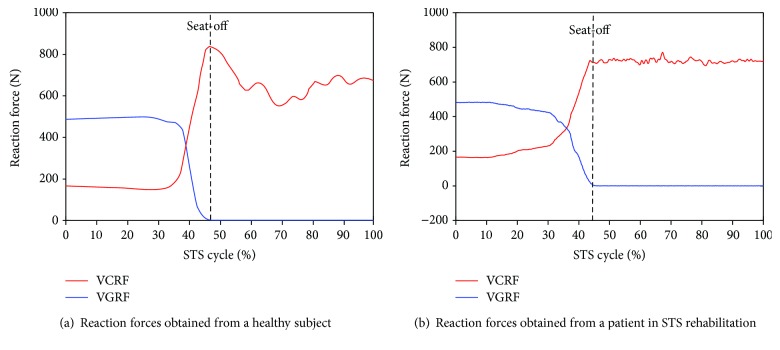
Typical VCRF from plate A (red line) under the feet and VGRF from force plate B (blue line) on the chair in the STS tasks performed by a healthy subject and a patient in STS rehabilitation.

All signals captured by the developed sensor system (IMUs and force plates) and the RCS were offline processed by Matlab. A low-pass filter with a cutoff frequency of 20 Hz was used to remove noise from all the raw data. A typical group of the calculated compositive hip, knee, and ankle joint angles (green, red, and blue lines correspondingly) and COM angular accelerations of the HAT, thigh, and shank (green, red, and blue lines correspondingly) derived from one of the five healthy subjects using the IMUs (dotted lines) and the RCS (solid lines) were compared as shown in Figure 4(a) and Figure 5(a), and the same parameters acquired from one of the five patients in STS rehabilitation were compared as shown in Figure 4(b) and Figure 5(b). The corresponding VCRF and VGRF in the same STS task performed by the healthy subject and the patient in STS rehabilitation were, respectively, shown in Figures 6(a) and 6(b), which were used to offer the referenced standard point (seat-off) within whole STS to synchronize the three trials of a task to the same percentage metric. The entire STS cycles of all the trials were synchronized by a percentage metric.

The accuracy of the developed sensor system should be evaluated by comparing the measured original inertial data (joint angular velocities and accelerations of the segments' COM) with those captured by the RCS, but not by comparing the joint moments, which were calculated from the original inertial data. However, since the previous calculated compositive joint angles (*θ*_1_, *θ*_2_, and *θ_3_*) and angular accelerations of each segment's COM (*α*_1_, *α*_2_, and *α*_3_) were derived from the synchronized ensemble averages of the measured original angular velocities and accelerations of three STS trails in a task, the accuracy of the sensor system could be more sufficiently evaluated by comparing *θ*_1_, *θ*_2_, *θ*_3_, *α*_1_, *α*_2_, and *α*_3_ based on more groups of measured original data between the two different systems. All the analysis parameters between the referenced and calculated compositive joint angles (*θ*_1_, *θ*_2_, and *θ*_3_) and angular accelerations of each segment's COM (*α*_1_, *α*_2_, and *α*_3_) of the five healthy subjects are, respectively, shown in Table 2 and Table 3, where RMSE was the root mean square of the differences between the referenced and calculated values, *R* was Pearson's correlation coefficients, and *e*_max_ was the maximum error. The same analysis parameters of the five patients are shown in Table 4 and Table 5. The two groups of the subjects in the four tables were numbered according to their STS times from short to long. The agreement between the data of the IMUs and RCS synchronized and normalized to the STS cycle was derived from *R*, which were categorized as weak (*R ≤* 0.35), moderate (0.35 < *R ≤* 0.67), strong (0.67 < *R ≤* 0.9), and excellent (*R* > 0.9), according to [26].

**Figure 7:**
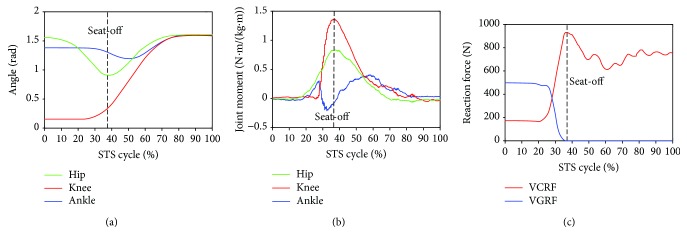
Percentage synchronized ensemble averages of the three joint angles (a), three joint moments (b), and two reaction forces (c) out of the 15 trials (3 trials × 1 task per subject × 5 healthy male subjects) as the reference (JMr) for comparing and analyzing the STS rehabilitation training of the five patients in rehabilitation.

**Figure 8:**
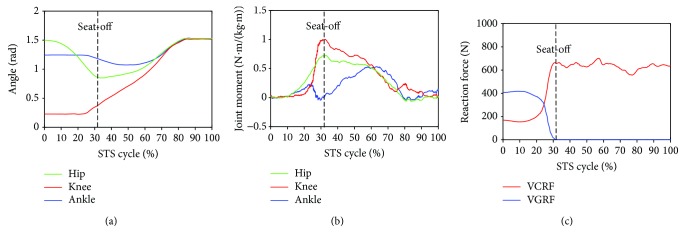
Percentage synchronized ensemble averages of the three joint angles (a), three joint moments (b) and two reaction forces (c) out of the 3 trials of a task performed by patient 1 (P1).

**Figure 9:**
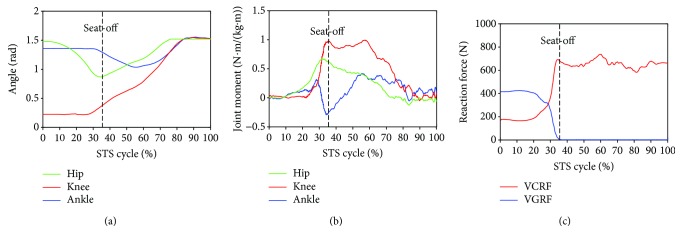
Percentage synchronized ensemble averages of the three joint angles (a), three joint moments (b), and two reaction force (c) out of the 3 trials of a task performed by patient 2 (P2).

**Figure 10:**
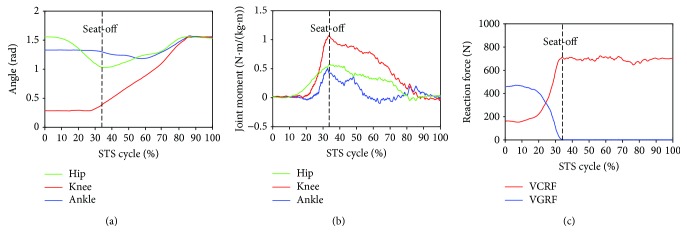
Percentage synchronized ensemble averages of the three joint angles (a), three joint moments (b), and two reaction force (c) out of the 3 trials of a task performed by patient 3 (P3).

**Figure 11:**
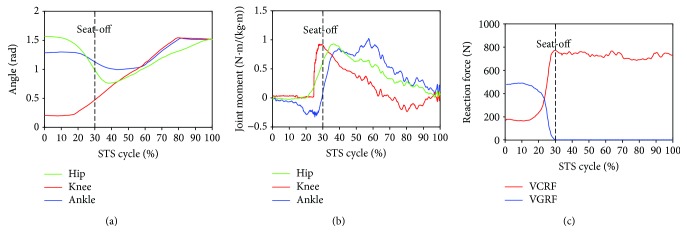
Percentage synchronized ensemble averages of the three joint angles (a), three joint moments (b), and two reaction force (c) out of the 3 trials of a task performed by patient 4 (P4).

**Figure 12:**
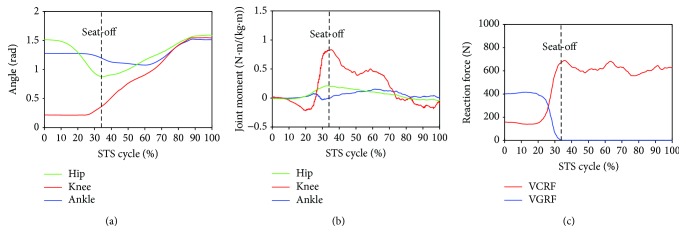
Percentage synchronized ensemble averages of the three joint angles (a), three joint moments (b), and two reaction force (c) out of the 3 trials of a task performed by patient 5 (P5).

**Figure 13:**
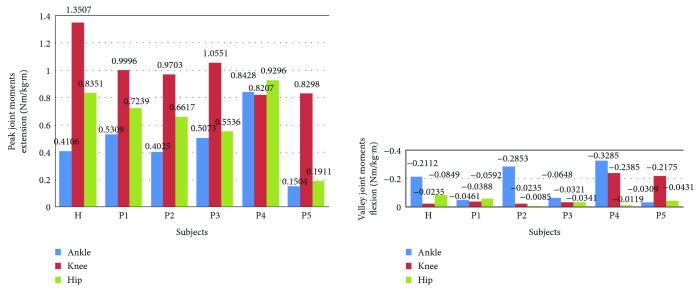
Comparison of the synchronized ensemble averages of the peak-valley joint moments in extension and flexion movements by the healthy subjects (H) and patients (P1, P2, P3, P4, and P5).

Furthermore, to analyze and evaluate the availability of the developed sensor system for noninvasively analyzing the kinetics of the STS movement, the joint moments of ankle, knee, and hip joints of the ten subjects were, respectively, calculated according to the presented method based on the corresponding synchronized ensemble averages of the measured original inertial data from IMUs. Thereinto, the five groups of hip, knee, and ankle joint moments of the five healthy subjects were further synchronized to get one group of ensemble average joint moments as the reference (JMr) for comparing and analyzing the STS rehabilitation training of the five patients and were shown in Figure 7. To compare the same joint moment of different STS tasks performed by different subjects, it was normalized to per Nm/kg·m divided by the height and mass of the corresponding subjects. All the corresponding curves of the patients were also shown in Figures 8–12. To do quantitative analysis of the STS movement, the peak-valley value of JMr and patient's joint moments (JMp), the peak value of VGRF and VCRF, as crucial quantitative characteristics, were shown in Table 6 and compared in Figure 13. Finally, to compare and analyze the STS rehabilitation status of each patient, the agreement between JMr and JMp synchronized to the STS cycle referred to the RSP was also derived from Pearson's correlation coefficients (*R*) and shown in Table 7.

## 5. Discussion and Conclusion

Comparing the same kinematic parameters (*θ*, *α*) derived from the developed IMUs (dotted lines) and the RCS (solid lines) curved by the same color but different lines in the same figure in Figure 4 and Figure 5, it was found that the corresponding parameters were basically the same except for a few subtle differences. Because the shape of each lower limb segment was an irregular approximate cone-cylinder, although a sensor-to-segment calibration procedure to relate the sensor orientations to segment orientations is performed, it was difficult to guarantee two axes of the IMUs absolutely in the sagittal plane in initial setting up. Therefore, the measured angular velocities and accelerations for calculating the joint angles, angular accelerations, and joint moments was not exactly the data needed in the equations but that with certain systematic errors and noise. Especially referred to all the analysis parameters between the referenced and calculated joint angles (*θ*_1_, *θ*_2_, and *θ*_3_) and angular accelerations of each segment's COM (*α*_1_, *α*_2_, and *α*_3_) of the five healthy subjects and five patients in Tables 2–5, faster and more fluent STS movements performed by either healthy subjects or patients resulted in lower *e*_max_ and RMSE and greater correlation coefficient. As it was more difficult to firmly fix the IMUs on the soft human body segments than on a rigid body without any slight movement, the errors were predictable and inevitable. Especially in lower-speed STS motion, long duration of skin motion artifact due to impact loading and muscle activation, body-sway motion in nonfluent STS trials would inevitably contaminate the measured original angular velocities and accelerations and then bring errors to the calculated joint angles and angular accelerations. Compared to the *e*_max_, RMSE, and *R* of the patients in rehabilitation and those of the healthy subjects, it suggests that the STS motion performed by the patients at a lower speed implicated more muscle tremble or body-sway motion which also contaminated the measured signal and resulted in greater *e*_max_ and RMSE and lower correlation coefficient.

The presented method used a rigid-body linked-segment model in which the positions of the end points and joints were estimated through predefined measured lengths and IMU-derived segment orientations. The moment of inertia of each segment, initial segment mass, segment lengths, and the position of COM were estimated or manually measured before each task individually as show in Table 1. Moreover, calibration limitations, such as a mismatch between the pose performed by the subject and the pose that the computational model is assuming, can cause errors. However, the results in Table 2 and Table 3 showed that the joint angles and angular accelerations derived from the IMUs were closed to those from the RCS with strong (0.67 < *R* ≤ 0.9) and even excellent correlation coefficient (all *R* > 0.93) by healthy subjects. It suggests that the developed wearable sensor system based on the presented method was available for noninvasive estimation of the kinematic parameters to calculate joint moments and do kinetic analysis of STS. Although the parameters in Tables 4 and 5 derived from the patients in STS rehabilitation was less satisfactory compared with the parameters derived from the healthy subjects with the least *R* = 0.522, it still was moderate and suggests that the developed wearable sensor system was more accurate to evaluate a more fluent and coherent STS movement performed by healthy subjects with sufficient muscle force, but it was also suitable for estimating the STS movement of a patient who can perform a complete STS task independently.

Comparing the two groups of typical joint angles of a healthy subject and a patient in Figure 4, it suggests that the HAT took action first in the three segments of both subjects. When the HAT swung forward without reaching the maximum hip joint angle, the knee joint angle had begun to increase (seat-off, 47% for healthy subjects, 45% for patients), then the HAT continued to move forward after the knee joint started to extend. However, the difference was that the HAT of the patients in rehabilitation swung more forward with greater hip joint angle than the healthy subjects. Because the inertia joint moments of the patients with lower STS speed was smaller than those of the healthy subjects with higher STS speed, the patients needed to adjust their center of gravity exactly above the feet with an exaggerated hip flexion strategy than the healthy subjects. The strategy could potentially compensate for the inability to generate lower extremity muscle force, so that the COM could be placed further ahead and the lower extremity moments could be redistributed potentially for easy STS. The ankle joint angles of both the healthy subject and the patient began to decrease first and then increase after seat-off. Larger moment of momentum of the HAT of the healthy subject would promote more obvious motion of the thigh and shank then resulted in exaggerated flexion angle of the ankle joint, while it was different in the patients in rehabilitation.

As shown in Figure 5, the angular accelerations of all segments' COMs during the STS trial performed by the patient varied less obviously and the peak value was also significantly less than those of the healthy subjects. It suggests that the patient in rehabilitation could not yet stand up in relative shorter time fluently and coherently as a healthy subject stood up with sufficient muscle force. And the lower limb muscles of the patients in rehabilitation still suffered activation trembles which contaminated the IMUs' signal and resulted in errors. Based on all the trials, if the STS movement was performed longer than 7 seconds, regarded as a quasistatic state, all the kinematic data measured by IMUs fluctuated even close to zero, which was almost of no availability. In this case, the joint moments resulted from the kinematic factors could be almost disregarded and it should be directly estimated with the moments contributed by the gravities of all the body segments. Therefore, the developed sensor system was almost inapplicable to estimate the joint moments of quasistatic STS movement.

Comparing the moments of the same joint of the healthy subjects (JMr) and patients (JMp) in Figures 7–12, it suggests that the inertia joint moment component played an important role in the resultant joint moment to contribute more fluent and successful STS movement for healthy subjects. That the knee joint moments (red line) of both the healthy subjects and patients had been increasing before seat-off suggests the existence of the static component of the knee joint moment. In other words, before the knee joint angle changed, the knee joint moments had already been increasing to react the gravity moment acting on the knee joint for preparing to sit up and stretch. Then the VCRF vanished and the VGRF rapidly reached the maximum after the subject leaved the chair (seat-off) and started to stretch.

Referring to Figures 8 and 9 derived from P1 and P2, the variation tendency of hip, knee, and ankle joint angles in the left charts of Figures 8 and 9 were similar to those of the healthy subjects in the left chart of Figure 7 suggesting that the proprioception on the STS posture and the STS balance control ability of the patients has recovered well in the rehabilitation training. But the exaggerated long duration hip flexion (green line in Figure 9(a)) led to a greater dorsiflexion of the ankle angle (blue line in Figure 9(a)) and a greater valley value of ankle joint moment (blue line in Figure 9(b)). After consulting with P2, he was weak to control the muscles of the thigh and experienced shakes of the thigh after seat-off, which proved the vibration of the knee joint moment after seat-off (red line in Figure 9(b)). Referring to Figures 10 and 12, derive *d* from P3 and P5; the dorsiflexion angles of ankle joints of P3 and P5 were not so obvious and the valley values of the ankle joint angle emerged later than those of other patients. The hip joint angle of P3 swung forward the least in the five patients. Meanwhile, almost no dorsiflexion moments appeared in the ankle joint of P3 and P5 before seat-off but stretched slowly with moderate hip joint moments. After consulting with P3 and P5, P3 reported that it was difficult to swing HAT forward enough with some spinal trauma, so that the hip joint moment was not great and the shank was almost not swung forward but just stretched after seat-off with dorsiflexion ankle joint moment; P5 reported to have difficulty in ankle joint that led to insufficient ankle joint moment for stretching off, so that he performed a low-speed STS movement with less swing of the shank (smaller ankle joint angle) and slow swing of HAT (smaller hip joint moment). Referring to Figure 11 derived from P4, the difference comparing Figure 7 derived from healthy subjects was that the HAT of P4 swung more forward with greater and later valley value of hip joint angle and with later peak value of hip joint moment after seat-off. Because P4's inertia knee joint moment at lower STS speed with leg muscle weakness was less than those of the healthy subjects, he needed to adjust his center of gravity exactly above the feet with an exaggerated hip flexion strategy than the healthy subjects. The strategy would cause a substantial increase of the plantar-flexion ankle joint moment (blue line in Figure 11(b)) and could potentially compensate for the inability to generate greater lower limb muscle force, so that the COM could be placed further ahead and the lower limb moments could be redistributed potentially for easy STS. Larger moment of momentum of the HAT of P4 promoted an obvious motion of the thigh and shank then resulted in exaggerated flexion angle of ankle joint (blue line in Figure 11(a)) and greater valley value of ankle joint moment before seat-off.

As shown in Table 6, peak-valley joint moments of the healthy subjects and patients were calculated from the data derived from the IMUs. The average peak knee extension moment was 1.3507 N·m·kg^−1^·m^−1^, the average peak hip extension moment was 0.8351 N·m·kg^−1^·m^−1^, and the average peak ankle plantar flexion moment was 0.4106 N·m·kg^−1^·m^−1^ for healthy subjects. It suggests that greatest joint moment of the hip, knee, and ankle joints was generated on the knee joint around seat-off. And it also suggests that the knee joint plays the most important role in STS and the phase around seat-off in the STS rehabilitation should be paid more attention. The comparison histogram of the synchronized ensemble averages of the peak-valley joint moments in extension and flexion movements by the healthy subjects and patients in Figure 13 was imaged and intuitively suggests the rehabilitation situations of the lower limb muscle maximum capacity in STS movement. The correlation coefficient of joint angles and moments of each patient compared with the references indicates the agreement of STS movement between the patients and the healthy subjects, which is valuable for evaluating the recovery of the whole STS rehabilitation training.

As the subjects in the experiment were limited to only five patients in rehabilitation and five healthy males, the results could not cover all cases of patients in STS rehabilitation training. Therefore, to verify systematic errors and measuring errors of the developed wearable sensor system for estimate joint moments of rehabilitation, more studies are necessary to determine reliability and validity of the system for more diverse subjects, especially for clinical populations. Since there was no integration of acceleration or angular velocity in the calculation of the joint angles and joint moments, the results were not distorted without considering drift errors. However, the results were still affected by offset errors by misalignment of the inertial sensors with the reference system; it was small but inevitable. Customized IMUs were used in the experiment, which could test angular velocities and accelerations about three orthogonal axes and packaged in a single SMT (1.1 × 1.1 × 0.50 inches) with rechargeable batteries that was convenient to wear for patients. Especially compared with the RCS of high cost and large space occupation, the developed wearable sensor system could provide adequate and necessary quantitative analysis of joint moments noninvasively. Another advantage of this method is that the developed device is not model-dependent which is very practical to continuously monitor the kinetic characteristics of patients in rehabilitation in home or to provide real-time feedback joint moments for the wearable powered exoskeleton assistant system. With the miniaturization of the inertial sensors, we are working to promote the developed wearable sensor and analysis systems to clinical applications.

Consequently, although the developed prototype of the wearable sensor system was only tested in ideal conditions in the lab with ten subjects, it provided a methodological reference for noninvasively evaluating functional rehabilitation state in STS dysfunction patients by kinetic analysis with the piecewise 2D three-segment STS biomechanical model. We innovatively analyzed both kinematics and kinetics of STS motion noninvasively with wearable sensor system, especially creatively estimated the lower limb joint moments with wearable inertial sensors for STS rehabilitation training analysis. The results showed insight into the movement coordination of STS and had implications for the ongoing development of more effective training techniques in the clinic.

## Figures and Tables

**Table 1:** Average inertia parameters of body segments of current Chinese male adults according to Chinese national standards.

Segments (definition)	Segment length/height (%)	Segment mass/whole body mass (%)	Center of mass/segment length distal	Moment of inertia (kg·m^2^)
Foot (lateral malleolus/head metatarsal)	14.77	3.6	0.5	0.0044
Shank (femoral condyles/medial malleolus)	23.86	10.6	0.567	0.0385
Thigh (greater trochanter/femoral condyles)	28.13	22.7	0.567	0.1978
HAT (greater trochanter/glenohumeral joint)	50.17	63.1	0.374	0.9180

**Table 2:** Analysis and comparison of compositive joint angles (*θ*_1_, *θ*_2_, and *θ*_3_) derived from the IMUs and RCS in STS tasks performed by healthy subjects.

Healthy subjects	Ankle joint angle *θ*_1_ (rad)			Knee joint angle *θ*_2_ (rad)			Hip joint angle *θ*_3_ (rad)		
	RMSE	*R*	*e*_max_	RMSE	*R*	*e*_max_	RMSE	*R*	*e*_max_
1	0.079	0.951	0.094	0.068	0.919	0.166	0.031	0.933	0.120
2	0.092	0.939	0.101	0.069	0.902	0.198	0.039	0.929	0.124
3	0.112	0.926	0.114	0.085	0.869	0.201	0.046	0.928	0.157
4	0.117	0.919	0.179	0.094	0.853	0.229	0.054	0.915	0.195
5	0.123	0.917	0.198	0.112	0.808	0.286	0.060	0.901	0.287
Average	0.105	0.930	0.137	0.087	0.870	0.216	0.046	0.921	0.177

**Table 3:** Analysis and comparison of compositive angular accelerations of each segment's COM (*α*_1_, *α*_2_, and *α_3_*) derived from the IMUs and RCS in STS tasks performed by healthy subjects.

Healthy subjects	Ankle joint angular acceleration *α*_1_ (rad/s^2^)			Knee joint angular acceleration *α*_2_ (rad/s^2^)			Hip joint angular acceleration *α*_3_ (rad/s^2^)		
	RMSE	*R*	*e*_max_	RMSE	*R*	*e*_max_	RMSE	*R*	*e*_max_
1	3.056	0.885	0.725	2.998	0.912	0.924	1.646	0.824	0.724
2	3.243	0.843	0.953	3.054	0.895	0.742	1.850	0.815	0.753
3	3.562	0.821	0.966	3.155	0.852	0.977	2.096	0.795	0.868
4	3.982	0.796	1.028	3.432	0.824	1.124	2.554	0.801	0.902
5	4.244	0.730	1.150	3.752	0.810	1.109	2.730	0.765	0.890
Average	3.617	0.815	0.964	3.278	0.859	0.975	2.175	0.800	0.827

**Table 4:** Analysis and comparison of compositive joint angles (*θ*_1_, *θ*_2_, and *θ*_3_) derived from the IMUs and RCS in STS tasks performed by patients.

Patients	Ankle joint angle *θ*_1_ (rad)			Knee joint angle *θ*_2_ (rad)			Hip joint angle *θ*_3_ (rad)		
	RMSE	*R*	*e*_max_	RMSE	*R*	*e*_max_	RMSE	*R*	*e*_max_
1	0.117	0.805	0.103	0.069	0.808	0.217	0.049	0.848	0.180
2	0.159	0.819	0.124	0.075	0.751	0.324	0.051	0.741	0.184
3	0.235	0.740	0.131	0.087	0.748	0.255	0.054	0.788	0.232
4	0.257	0.737	0.205	0.090	0.745	0.207	0.063	0.692	0.243
5	0.362	0.625	0.166	0.096	0.713	0.269	0.070	0.715	0.199
Average	0.226	0.745	0.146	0.083	0.753	0.254	0.057	0.757	0.208

**Table 5:** Analysis and comparison of compositive angular accelerations of each segment's COM (*α*_1_, *α*_2_, and *α*_3_) derived from the IMUs and RCS in STS tasks performed by patients.

Patients	Ankle joint angular acceleration *α*_1_ (rad/s^2^)			Knee joint angular acceleration *α*_2_ (rad/s^2^)			Hip joint angular acceleration *α*_3_ (rad/s^2^)		
	RMSE	*R*	*e*_max_	RMSE	*R*	*e*_max_	RMSE	*R*	*e*_max_
1	0.124	0.524	0.195	0.122	0.563	0.143	0.189	0.621	0.132
2	0.182	0.542	0.220	0.147	0.522	0.213	0.201	0.608	0.237
3	0.167	0.601	0.172	0.261	0.512	0.249	0.227	0.534	0.258
4	0.201	0.491	0.253	0.166	0.509	0.197	0.186	0.522	0.355
5	0.230	0.483	0.279	0.245	0.504	0.288	0.223	0.499	0.306
Average	0.181	0.528	0.224	0.188	0.522	0.218	0.205	0.557	0.258

**Table 6:** Kinetic analysis based on the data derived from the healthy subjects and patients using the developed wearable sensor system.

Synchronized ensemble averages		Healthy subjects	Patient 1	Patient 2	Patient 3	Patient 4	Patient 5
Peak-valley joint moments (Nm/kg·m)	Ankle dorsiflexion	−0.2112	−0.0462	−0.2853	−0.0648	−0.3285	−0.0309
	Ankle plantarflexion	0.4106	0.5309	0.4025	0.5073	0.8428	0.1504
	Knee flexion	−0.0235	−0.0388	−0.0235	−0.0321	−0.2385	−0.2175
	Knee extension	1.3507	0.9996	0.9703	1.0551	0.8207	0.8298
	Hip flexion	−0.0849	−0.0592	−0.0085	−0.0341	−0.0119	−0.0431
	Hip extension	0.8351	0.7239	0.6617	0.5536	0.9296	0.1911

Peak force (N)	VGRF	930.7	635.5	720.3	703.2	761.3	671.1
	VCRF	499.7	419.8	420.9	482.9	485.6	420.9

**Table 7:** The correlation coefficient of joint angles and moments of each patient compared with the reference.

Parameters		*R* of the patients				
		*R*_1_	*R*_2_	*R*_3_	*R*_4_	*R*_5_
Angles	Hip (*θ*_1_)	0.8521	0.6133	0.5970	0.4428	0.5054
	Knee (*θ*_2_)	0.5257	0.4319	0.4720	0.3168	0.4722
	Ankle (*θ*_3_)	0.8071	0.7758	0.6943	0.5480	0.6012

Moments	Hip (*M*_3_)	0.3001	0.2965	0.3546	0.2219	0.0904
	Knee (*M*_2_)	0.2909	0.2725	0.1073	0.1928	0.1050
	Ankle (*M*_1_)	0.3460	0.2853	0.0648	0.1825	0.0309

## Notations

${\vec{M}}_{i}$: joint moment vector about joint *i* (*i* = 1, 2, 3; ankle joint, knee joint, and hip joint)
*J*_*j*_: moment of inertia of segment *j* about the center of mass (*j* = 1, 2, 3; shank, thigh, and HAT)
${\vec{\alpha }}_{j}$: actual angular acceleration vector of segment *j* about the center of mass, containing the acceleration of the center of gravity
${\vec{r}}_{ij}$: position vector from joint *i* to the center of gravity of segment *j*
*m*_*j*_: mass of segment *j*
*k*_*j*_: position of the COM of the segment *j*
${\vec{a}}_{j}$: acceleration vector of the center of gravity of segment *j*
*g*: acceleration of the center of gravity
*T*_com_: STS trajectory of the COM
*θ*_*i*_: angle of joint *i* (*i* = 1, 2, 3; ankle joint, knee joint, and hip joint)
${\vec{F}}_{hip}$: is the equivalent external force acting on the hip joint from the chair.
